# Supplementary figures and images for: Potential of Korean forest tree seed extracts as multifunctional bioresources: Evaluation of Antioxidant, anti-inflammatory, whitening, and anticancer activities
Source: PLoS One. 2026 Jul 27;21(7):e0345845. doi: 10.1371/journal.pone.0345845 (PMC13405096; doi:10.1371/journal.pone.0345845)

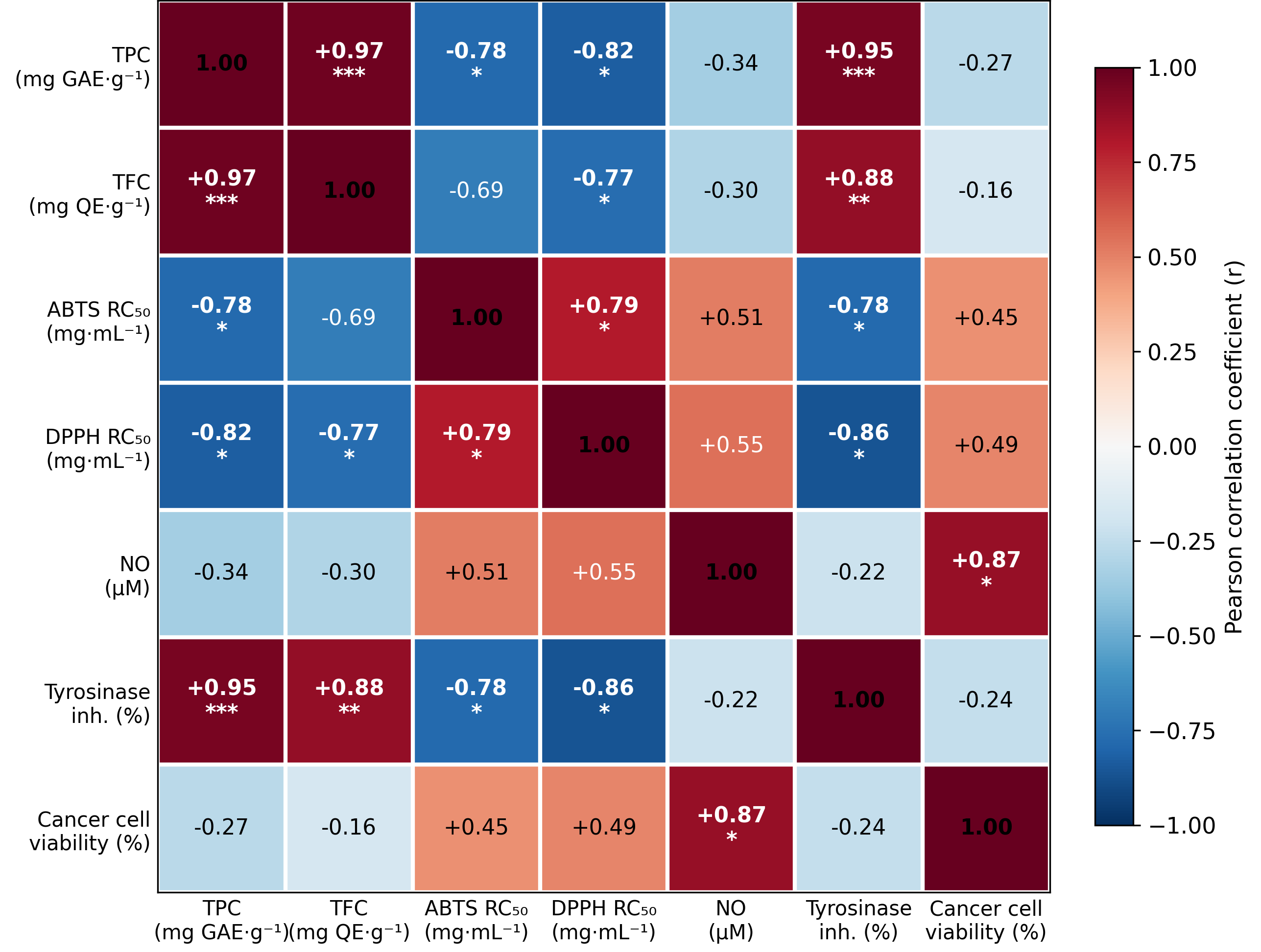

Supplement: S1 Fig — Pearson correlation coefficients r were calculated using species-level mean values (n = 7) for TPC, TFC, ABTS, DPPH, NO concentration, tyrosinase inhibition, and mean cancer cell viability. Asterisks indicate statistical significance (* p < 0.005, ** p < 0.01, *** p < 0.001). (TIF) [file pone.0345845.s001.tif]
